# Machine learning algorithms trained with pre-hospital acquired history-taking data can accurately differentiate diagnoses in patients with hip complaints

**DOI:** 10.1080/17453674.2021.1884408

**Published:** 2021-02-12

**Authors:** Michiel Siebelt, Dirk Das, Amber Van Den Moosdijk, Tristan Warren, Peter Van Der Putten, Walter Van Der Weegen

**Affiliations:** a Department of Orthopedic Surgery, St Anna Hospital, Geldrop;; b Leiden Institute of Advanced Computer Science, Leiden University Leiden, The Netherlands

## Abstract

Background and purpose — Machine learning (ML) techniques are a form of artificial intelligence able to analyze big data. Analyzing the outcome of (digital) questionnaires, ML might recognize different patterns in answers that might relate to different types of pathology. With this study, we investigated the proof-of-principle of ML-based diagnosis in patients with hip complaints using a digital questionnaire and the Kellgren and Lawrence (KL) osteoarthritis score.

Patients and methods — 548 patients (> 55 years old) scheduled for consultation of hip complaints were asked to participate in this study and fill in an online questionnaire. Our questionnaire consists of 27 questions related to general history-taking and validated patient-related outcome measures (Oxford Hip Score and a Numeric Rating Scale for pain). 336 fully completed questionnaires were related to their classified diagnosis (either hip osteoarthritis, bursitis or tendinitis, or other pathology). Different AI techniques were used to relate questionnaire outcome and hip diagnoses. Resulting area under the curve (AUC) and classification accuracy (CA) are reported to identify the best scoring AI model. The accuracy of different ML models was compared using questionnaire outcome with and without radiologic KL scores for degree of osteoarthritis.

Results — The most accurate ML model for diagnosis of patients with hip complaints was the Random Forest model (AUC 82%, 95% CI 0.78–0.86; CA 69%, CI 0.64–0.74) and most accurate analysis with addition of KL scores was with a Support Vector Machine model (AUC 89%, CI 0.86–0.92; CA 83%, CI 0.79–0.87).

Interpretation — Analysis of self-reported online questionnaires related to hip complaints can differentiate between basic hip pathologies. The addition of radiological scores for osteoarthritis further improves these outcomes.

Use of artificial intelligence (AI) techniques like data mining, machine learning (ML), and deep learning are now starting to erupt within healthcare, with first applications aimed at cancer diagnostics (Nguyen et al. 2018, Codari et al. 2019), cardiology (Nirschl et al. 2018) and image recognition in radiology (Wang et al. 2017, Fourcade and Khonsari 2019).

AI is also emerging within the field of orthopedic surgery (Duffield et al. 2017). Earlier work using AI in orthopedic studies showed the ability of ML to classify knee osteoarthritis (OA) subjects versus healthy patients. Based on kinematic data Kotti et al. (2017) achieved an accuracy of 73%. In comparison with that study, which collected its data in a laboratory setting, Dolatabadi et al. (2017) used kinematic data from more unobtrusive sensors and were also able to distinguish OA subjects from healthy patients. Other ML-related publications in orthopedics report on spine pathology detection, fracture detection, and bone and cartilage image segmentation (Ashinsky et al. 2015).

However, to our knowledge, no studies in orthopedics have developed ML algorithms for predicting a clinical diagnosis. In this paper we used information from digital intake forms, which were completed online by our patients before initial consultation with an orthopedic surgeon. We sought to determine (1) the accuracy of different ML algorithms to predict a pre-hospital diagnosis in patients suffering from hip complaints based on history-taking questions only, and (2) how much radiographic imaging results contribute to accurately predicting a diagnosis in these patients.

## Patients and methods

For the development of an ML algorithm we designed a prospective cohort study that included patient data from a single hospital (St Anna Hospital, Geldrop, The Netherlands).

All patients aged > 55 years with hip complaints were eligible for inclusion. Immediately after contacting the hospital to schedule an appointment, all participating patients received our questionnaire by e-mail, which had a hyperlink embedded, leading to a secure online environment (Interactive Studios, Rosmalen, the Netherlands). Here, patients were able to answer all questions before initial consultation, which was usually within 1 to 2 weeks. For this purpose we used our online patient reported outcome measurement (PROM) system. This system is normally used to collect standardized PROMs before and after surgery to track orthopedic healthcare outcomes from a patient’s perspective. Within this online environment, 2 authors (MS and WvdW) created a new questionnaire for the purpose of this study, which was verified by a third author (DD).

This new questionnaire included standard history-taking questions for suspected hip pathology (i.e., location of pain, severity and duration of symptoms; an overview of all questions is presented in Supplementary data 1—complete questionnaire). These questions were combined with well-validated PROM questionnaires: the Oxford Hip Score (OHS) (Dawson et al. 1996, de Groot et al. 2007), and severity of pain measured with a Numeric Rating Scale (NRS) (Salaffi et al. 2004). Questionnaires of patients who responded to our digital intake form were checked. Incomplete questionnaires were excluded, except for missing answers in the Oxford Hip Score (OHS). As advocated for this specific hip score, a maximum of 2 missing items is allowed and can be dealt with by replacing missing scores with the average score of completed items (Dawson et al. 1996, Murray et al. 2007).

After history taking, physical examination and radiographic evaluation, all patients were informed of their diagnosis by their consulting orthopedic surgeon. We retrieved this diagnosis from the medical file and linked it to the questionnaire for that specific patient. This diagnosis was assigned to 1 of 3 categorical outcomes: (1) osteoarthritis (OA) of the hip; (2) bursitis or tendinitis around the hip; or defined as (3) other pathology. These 3 diagnoses were chosen since they represent a large portion of hip complaints. For this proof-of-principle we did not want to start with more diagnoses, since ML techniques will have more trouble differentiating between many possible outcome options and therefore would require larger numbers of patient-reported questionnaires.

This dataset was imported into Orange Workflow (version 3.22, Ljubljana, Slovenia), which is an open-source AI software system using different ML techniques. Using Orange, a data file was created to train and test the algorithms in a 10-fold stratified cross-validation loop. The 27 variables were ranked for their ability to differentiate between the 3 diagnosis groups by averaging the outcomes of multiple ranking techniques (Information Gain, Information Gain Ratio, Gini Decrease, X2, ReliefF, and Fast Correlation Based Filter [FCBF]). We trained and tested all ML models available in Orange Workflow (Constant, CN2 rule induces, k Nearest Neighbour [kNN], Tree, Random Forest, Support Vector Machine (SVM), Logistic Regression, Naïve Bayes, AdaBoost, and Neural Network). Orange does not have hyperparameter tuning capabilities so hyperparameters were selected by hand (for an overview of the hyperparameters see Table 1). Resulting area under the curve (AUC) and classification accuracy (CA) outcomes were used to identify the best scoring model (Duffield et al. 2017). 95% confidence intervals (CI) were calculated for AUC and AC with each ML model.

**Table 1.** Selected hyperparameters for each evaluated algorithm

Algorithm	Hyperparameter	Value
SVM	Epsilon	0.1
Cost (C)	1	
Kernel	RBF	
Decision tree	Min. number of	
instances in leaves	2	
Do not split subsets smaller than	5	
Max. tree depth	100	
Logistic regression	Regularization	L2
Cost (C)	1	
Neural network	Hidden neurons	100
Activation	ReLu	
Solver	Adam	
Alpha	0.0001	
KNN	Number of neighbors	5
Metric	Euclidean	
Weight	Uniform	
Random Forest	Number of trees	10
Number of attributes		
considered at each split	5	
Max. tree depth	3	
Do not split subsets smaller than	5	

Each model was first trained and evaluated on the questionnaire data with all questions included. Next, we investigated the possibility of achieving similar performance with fewer questions included in the dataset. For this purpose we evaluated the performance of predictive models that were trained on data only including the top 5 ranking questions, and in a second experiment only including the top 10 ranking questions. To analyze the contribution of radiographic imaging results to the diagnosis process, we scored the pelvic radiograph for each included patient using the Kellgren–Lawrence (KL) scoring method (Kellgren and Lawrence 1957) and trained and tested the algorithms again with this score KL added to the full dataset. With inclusion of KL scores, the model was again retested with all questions of the questionnaire, with only the top 5 questions and using only the top 10 questions.

### Ethics, registration, funding, and potential conflicts of interest

This study was reviewed by the regional medical ethical committee and was considered to be exempt from full review (registration number N19.066) according to Dutch law. The study protocol was registered in the Dutch Trial Register (Trial registration number NL8229). No external funding was obtained. The authors report no conflicts of interest.

**Figure:**
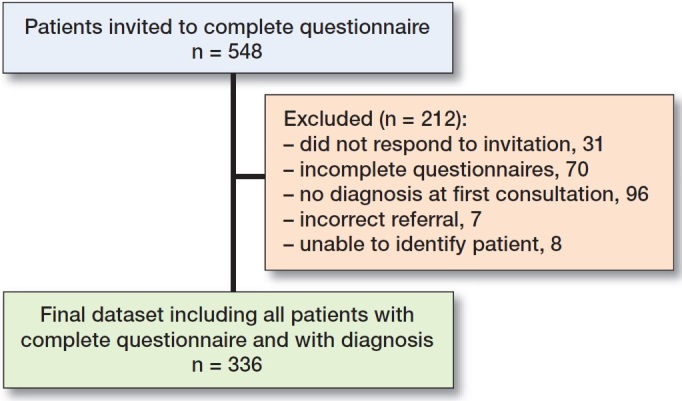
Study flow.

## Results

Questionnaires of 517 participating patients were received, but after checking for completeness of answers 336 patients could be included in the study (Figure). The collection of this data resulted in a dataset with 283 observations of 27 variables from the questionnaire (see Supplementary material) and 1 target variable (diagnosis group). The distribution of the target variables is as follows. 191 (68%) patients were diagnosed with OA, 61 (22%) patients were diagnosed with bursitis or tendinitis around the hip, and 31 (11%) were diagnosed with other pathology. There is a clear imbalance in the distribution of the target variables with OA being the overrepresented class. The Random Forest algorithm with 20 folds trained on the full dataset (all answers to all questions included) resulted in the highest AUC (82%, CI 0.78–0.86) and CA (69%, CI 0.64–0.74). The 5 most differentiating questions were (in decreasing order of differentiating power):

1. OHS 4: Have you been able to put on a pair of socks, stockings, or tights?
2. Do you experience pain in the groin area?
3. Does your hip feel stiff during the first steps you take when walking?
4. Would you be willing to undergo surgery if needed?
5. OHS 7: Are you able to walk up and down the stairs?

All ML models were tested to see if the AUC and/or CA improved by leaving out possibly less important questions. Using only the top 5 questions in the training set, logistic regression (10 folds) gave the highest results of 81% AUC (CI 0.77–0.85) and 73% CA (CI 0.67–0.77). When we selected the top 10 ranking questions, Neural Network (10 folds) gave the highest results with 74% AUC (CI 0.69–0.78) and 67% CA (CI 0.62–0.72) (Table 2). Adding the radiographic data to the dataset increased both the AUC and the CA of the ML models. The distribution of the KL scores accross the 3 diagnosis groups is presented in Table 3. Under this condition, SVM resulted in the highest AUC and CA scores of 89% (CI 0.86–0.92) and 83% (CI 0.79–0.87) respectively (Table 2). A full overview of all ML algorithms is described in Supplementary data.

**Table 2.** Artificial intelligence analysis using machine learning (ML) algorithms on pre-hospital-acquired patient history-taking form for patients aged > 55 years with hip complaints. Values are ML algorithm accuracy in percent

	History-taking only			KL score added		
			Prediction			Prediction
Dataset	AUC	CA	model	AUC	CA	model
All questions	82	69	RF	89	83	SVM
Top 5 questions only	82	73	SVM	85	79	SVM
Top 10 questions only	78	70	SVM	79	79	SVM

RF = Random Forest.

SVM = Standard Vector Machine.

**Table 3.** Distribution (%) of the KL scores accross the 3 diagnosis groups

	KL score				
Diagnosis	0	1	2	3	4
Bursitis/tendinitis	24	55	20	1	0
Osteoarthritis	1	7	26	48	18
Other	11	52	11	15	11

## Discussion

Computer algorithms which use patients’ answers to digital history-taking questions are capable of differentiating a hip complaint related diagnosis with fairly good accuracy (AUC 74% and CA 67%). Adding radiographic information results in even higher accuracy and improves AI performance (AUC 89% and CA 83%). Obviously, there are clear logistical problems that need to be solved in order to achieve integration of conventional radiological examination into pre-hospital AI analysis, but this study shows the proof-of-principle for ML techniques in orthopedics.

Our approach using ML may help improve patient care in many ways. With accurate prediction of diagnosis and related treatment, patients can be educated about their condition in advance of their hospital visit, which is easily managed by using a smartphone app. Such an app with supporting information may help patients to increase knowledge and understanding of underlying hip pathology related to their complaint. Subsequently, patients might experience a more in-depth first consultation with the orthopedic surgeon during their hospital visit (Timmers et al. 2018). In order to test this hypothesis, we are currently enrolling a prospective randomized controlled trial using pre-hospital AI diagnosis and its effect on patient knowledge and satisfaction levels during hospital consultation.

Besides patient satisfaction, ML diagnosis may also increase outpatient clinic efficiency. First, patients who are more likely to have a diagnosis that is treated nonoperatively can be grouped together when outpatient clinic appointments are scheduled. Other supporting healthcare providers (i.e., physician assistants, physical therapists) can be scheduled to join these consultations and patients’ complaints may be dealt with by a multidisciplinary team. Second, patients who are more likely to be treated with surgery (e.g., hip arthroplasty) can also be grouped together and simultaneously planned for preoperative screening, reducing the number of visits needed to the hospital to a minimum.

This predictive analytic study has several limitations. Most importantly, our questionnaire is of course in need of validation in other hospitals. Next, we grouped multiple hip pathologies in 3 categorical groups. This does not cover clinical reality in which orthopedic surgeons make a much more detailed diagnosis. Since this study is a first exploration of ML applied to the clinical diagnostic process in orthopedic surgery and history-taking in particular, we consider our approach justifiable for now. Larger datasets should allow further explorations using more detailed diagnostic outcomes.

Furthermore, our resulting accuracy of 82% is high, but could be insufficient in daily clinical practice since it still results in approximately 2 out of 10 patients receiving an incorrect diagnosis. The most important consideration is related to the number of patients receiving a wrong prediction (either a false-positive or a false-negative prediction. However, these computer algorithms should not be considered a substitute for the diagnostic process, but rather an aid to educate patients pre-hospital and organize outpatient clinic logistics.

In conclusion, ML algorithms are capable of making a clinical diagnosis for selected patients who suffer from hip complaints using online questionnaires. This first study yields an accuracy of 82% using outcome from our digital questionnaire only, which improved to 89% in combination with radiological osteoarthritis scores. Consultation of patients with complaints using ML techniques can therefore be considered as a valuable tool to aid the orthopedic surgeon in many practical ways, but should not yet be considered as a substitution for human made diagnosis.

## Supplementary Material

Supplemental Material

## Supplementary data

The complete questionnaire and overview of ML algorithms are available as supplementary data in the online version of this article, http://dx.doi.org/10.1080/17453674.2021.1884408

## References

[CIT0001] Ashinsky B G, Coletta C E, Bouhrara M, Lukas V A, Boyle J M, Reiter D A, Neu C P, Goldberg I G, Spencer R G. Machine learning classification of OARSI-scored human articular cartilage using magnetic resonance imaging. Osteoarthritis Cartilage 2015; 23: 1704–12. 26067517 10.1016/j.joca.2015.05.028 PMC4577440

[CIT0002] Codari M, Schiaffino S, Sardanelli F, Trimboli R M. Artificial intelligence for breast MRI in 2008–2018: a systematic mapping review. Am J Roentgenol 2019; 212: 280–92. 30601029 10.2214/AJR.18.20389

[CIT0003] Dawson J, Fitzpatrick R, Carr A, Murray D. Questionnaire on the perceptions of patients about total hip replacement. J Bone Joint Surg Br 1996; 78(2): 185–90. 8666621

[CIT0004] De Groot I B, Reijman M, Terwee C B, Bierma-Zeinstra S M A, Favejee M, Roos E M, Verhaar J A. Validation of the Dutch version of the Hip disability and Osteoarthritis Outcome score. Osteoarthritis Cartilage 2007; 15(1): 104–9. 16890460 10.1016/j.joca.2006.06.014

[CIT0005] Dolatabadi E, Taati B, Mihailidis A. An automated classification of pathological gait using unobtrusive sensing technology. EEE Trans Neural Syst Rehabil Eng 2017; 25: 2336–46. 10.1109/TNSRE.2017.2736939 28792901

[CIT0006] Duffield S J, Ellis B M, Goodson N, Walker-Bone K, Conaghan P G, Margham T, Loftis T. The contribution of musculoskeletal disorders in multimorbidity: implications for practice and policy. Best Pract Res Clin Rheumatol 2017; 31: 129–44. 29224692 10.1016/j.berh.2017.09.004

[CIT0007] Fourcade A, Khonsari R H. Deep learning in medical image analysis: a third eye for doctors. J Stomatol Oral Maxillofac Surg 2019; 120: 279–88. 31254638 10.1016/j.jormas.2019.06.002

[CIT0008] Kellgren J H, Lawrence J S. Radiological assessment of osteo-arthrosis. Ann Rheum Dis 1957; 16: 494–502. 13498604 10.1136/ard.16.4.494 PMC1006995

[CIT0009] Kotti M, Duffell L D, Faisal A A, McGregor A H. Detecting knee osteoarthritis and its discriminating parameters using random forests. Med Eng Phys 2017; 43: 19–29. 28242181 10.1016/j.medengphy.2017.02.004 PMC5390773

[CIT0010] Murray D W, Fitzpatrick R, Rogers K, Pandit H, Beard D J, Carr A J, Dawson J. The us of the Oxford hip and knee scores. J Bone Joint Surg Br 2007; 89(8): 1010–14. 17785736 10.1302/0301-620X.89B8.19424

[CIT0011] Nguyen A V, Blears E E, Ross E, Lall R R, Ortega-Barnett J. Machine learning applications for the differentiation of primary central nervous system lymphoma from glioblastoma on imaging: a systematic review and meta-analysis. Neurosurg Focus 2018; 45: E5. 10.3171/2018.8.FOCUS18325 30453459

[CIT0012] Nirschl J J, Janowczyk A, Peyster E G, Frank R, Margulies K B, Feldman M D, Madabhushi A. A deep-learning classifier identifies patients with clinical heart failure using whole-slide images of H&E tissue. PLOS ONE 2018; 13: e0192726. 29614076 10.1371/journal.pone.0192726 PMC5882098

[CIT0013] Salaffi F, Stancati A, Silvestri C A, Ciapetti A, Grassi W. Minimal clinically important changes in chronic musculoskeletal pain intensity measured on a numerical rating scale. Eur J Pain 2004; 8(4): 283–91 15207508 10.1016/j.ejpain.2003.09.004

[CIT0014] Timmers T, Janssen L, Pronk Y, van der Zwaard B C, Koëter S, van Oostveen D, de Boer S, Kremers K, Rutten S, Das D, van Geenen RC, Koenraadt K L, Kusters R, van der Weegen W. Assessing the efficacy of an educational smartphone or tablet app with subdivided and interactive content to increase patients’ medical knowledge: randomized controlled trial. JMIR Mhealth Uhealth 2018; 6: e10742. 30578185 10.2196/10742 PMC6320423

[CIT0015] Wang X, Peng Y, Lu L, Lu Z, Bagheri M, Summers R M. ChestX-ray8: hospital-scale chest X-ray database and benchmarks on weakly-supervised classification and localization of common thorax diseases. 2017 IEEE Conference on Computer Vision and Pattern Recognition (CVPR), Honolulu, HI; 2017. pp 3462–71.

