# Supplementary material for: Machine learning algorithms trained with pre-hospital acquired history-taking data can accurately differentiate diagnoses in patients with hip complaints
Source: Acta Orthop. 2021 Feb 12;92(3):254–7. doi: 10.1080/17453674.2021.1884408 (PMC8231380; doi:10.1080/17453674.2021.1884408)
Supplement: Supplemental Material [file IORT_A_1884408_SM7382.pdf]

Supplementary data

The complete questionnaire

Please answer the following questions regarding your hip complaints

Q(uestion) 1. Pain while resting. Within a range from 0–10, please describe your maximal hip pain while resting. 0 = no pain, 10 = worst pain imaginable.
A(nswer) 1. 0, 1, 2, 3, 4, 5, 6, 7, 8. 9 or 10

Q2. Where do you experience the most hip pain?
A2. Over the front of the upper leg
Over the side of the upper leg
Within the groin
Over the buttock and backside of the upper leg

Q3. Pain while walking. Within a range from 0–10, please describe your maximal hip pain during walking. 0 = no pain, 10 = worst pain imaginable.
A3. 0, 1, 2, 3, 4, 5, 6, 7, 8. 9 or 10

Q4. Describe the progression of your complaint, your hip complaint:
A4. Has gotten worse
Remained the same
Has improved
Varies in severity, sometimes there are no complaints, sometimes it is worse.

Q5. For how long have you suffered from your hip complaint?
A5. 0–3 months
4–6 months
6–12 months
> 12 months

Q6. During first strides while walking, does your hip feel stiff?
A6. Yes
Sometimes
No

Q7. Because of your hip complaint, have you stopped working, competing in sports or other hobbies?
A7. Yes, I cannot participate in work, sports and hobbiess anymore
Yes, both hobbies and sports
Yes, I stopped participating in sports
No, but due to my complaint, I cannot perform to full extent
No, I participate in these activities without problems

Q8. Do you use painkillers for your complaints?
A8. Yes/No

Q9. What is your weight?
A9. Answer in kilograms:

Q10. What is your height?
A10. Answer in centimeters:

Q11. If a surgical procedure is necessary, would you be willing to undergo surgery?
A11. Yes
Maybe
No

Oxford Hip Score
During the past 4 weeks….

Q12. How would you describe the pain you usually have in your hip?
A12. None
Very mild
Mild
Moderate
Severe

Q13. Have you been troubled by pain from your hip in bed at night?
A13. No nights
Only 1 or 2 nights
Some nights
Most nights
Every night

Q14. Have you had any sudden, severe pain (shooting, stabbing, or spasms) from your affected hip?
A14. No days
Only 1 or 2 days
Some days
Most days
Every day

Q15. Have you been limping when walking because of your hip?
A15. Rarely/never
Sometimes or just at first
Often, not just at first
Most of the time
All of the time

Q16. For how long have you been able to walk before the pain in your hip becomes severe (with or without a walking aid)?
A16. No pain for 30 minutes or more
16 to 30 minutes
5 to 15 minutes
Around the house only
Not at all

Q17. Have you been able to climb a flight of stairs?
A17. Yes, easily
With little difficulty
With moderate difficulty
With extreme difficulty
No, impossible

Q18. Have you been able to put on a pair of socks, stockings, or tights?
A18. Yes, easily
With little difficulty
With moderate difficulty
With extreme difficulty
No, impossible

Q19. After a meal (sat at a table), how painful has it been for you to stand up from a chair because of your hip?
A19. Not at all painful
Slightly painful
Moderately painful
Very painful
Unbearable

Q20. Have you had any trouble getting in and out of a car or using public transportation because of your hip?
A20. No trouble at all
Very little trouble
Moderate trouble
Extreme difficulty
Impossible to do

Q21. Have you had any trouble with washing and drying yourself (all over) because of your hip?
A21. No trouble at all
Very little trouble
Moderate trouble
Extreme difficulty
Impossible to do

Q22. Could you do the household shopping on your own?
A22. Yes, easily
With little difficulty
With moderate difficulty
With extreme difficulty
No, impossible

Q23. How much has pain from your hip interfered with your usual work, including housework?
A23. Not at all
A little bit
Moderately
Greatly
Totally

Supplementary overview of ML models' results

Model	AUC (95% CI)	CA (95% CI)
Results without KL score		
Random Forest	82.2 (0.78–0.86)	69.4 (0.64–0.74)
Standard vector machine	82.1 (0.78–0.86)	62.1 (0.57–0.67
Neural network	76.4 (0.72–0.81)	69.7 (0.65–0.75)
AdaBoost	74.6 (0.70–0.79)	59.1 (0.54–0.64)
Naive Bayes	73.0 (0.68–0.78)	69.7 (0.65–0.75)
CN2 Rule Inducer	64.7 (0.60–0.70)	62.1 (0.57–0.67)
Tree	55.5 (0.50–0.61)	53.0 (0.48–0.58)
Constant	50.0 (0.45–0.55)	74.2 (0.70–0.79)
kNN	45.3 (0.40–0.51)	56.1 (0.51–0.61)
Results without KL score added		
Support vector machine	89.1 (0.86–0.92)	83.3 (0.79–0.87)
Random Forest	84.0 (0.80–0.88)	73.2 (0.68–0.78)
Neural network	82.2 (0.78–0.86)	75.0 (0.70–0.80)
Naive Bayes	80.1 (0.76–0.84)	53.6 (0.48–0.59)
CN2 Rule Inducer	77.8 (0.73–0.82)	64.3 (0.59–0.69)
Tree	74.9 (0.70–0.80)	66.1 (0.61–0.71)
Adaboost	68.6 (0.64–0.74)	69.6 (0.65–0.75)
kNN	60.6 (0.55–0.66)	66.1 (0.61–0.71)
Constant	50.0 (0.45–0.55)	69.6 (0.65–0.75)
